# A Systematic Review on the Use of Aspirin in the Prevention of Deep Vein Thrombosis in Major Elective Lower Limb Orthopedic Surgery: An Update from the Past 3 Years

**DOI:** 10.1055/s-0037-1615817

**Published:** 2017-12-29

**Authors:** Dylan A. Mistry, Amit Chandratreya, Paul Y. F. Lee

**Affiliations:** 1 South Wales Orthopaedic Research Network, Cardiff University, Welshbone, Cardiff, United Kingdom; 2 Grantham and District Hospital, United Lincolnshire Hospitals NHS Trust, Lincoln, United Kingdom; 3 Department of Trauma and Orthopaedic, LEO Institute, Grantham, United Kingdom; 4 Princess of Wales Hospital, Abertawe Bro Morgannwg University Health Board, Bridgend, United Kingdom

**Keywords:** aspirin, anticoagulants, venous thromboembolism, prophylaxis, deep vein thrombosis

## Abstract

**Introduction** Currently there are no consensuses in the national guidance on thromboprophylaxis following major elective lower limb surgery. Emerging clinical evidence suggests that aspirin could be just as effective as anticoagulants with a lower cost. The aim of this study was to provide an update based on literature of the past 3 years for the use of aspirin as thromboprophylaxis after knee and hip arthroplasty.

**Materials and Methods** MEDLINE/EMBASE search was performed with appropriate terms for original articles from 2014 to 2017.

**Results** Eight articles were found. Five articles concluded that aspirin was an effective prophylactic. The collation of results on the deep vein thrombosis rate involved 43,012 patients who were prescribed aspirin, of which 283 (0.66%) suffered from symptomatic deep vein thromboses. Aspirin was noted for its good side effect profile and cost effectiveness. It was noted that anticoagulants had a higher rate of complications, including bleeding and wound-oozing.

**Conclusion** Aspirin is an effective and safe prophylactic against deep vein thrombosis following major elective lower limb arthroplasty surgery.

Certain orthopedic surgeries, such as joint arthroplasty surgery, carry a substantial risk of venous thromboembolisms (VTEs). 1 2 Rates of deep vein thrombosis (DVT) in total knee arthroplasty can be as high as 88% when untreated (including asymptomatic patients); 3 however, when treated with prophylaxis, the rate of symptomatic DVTs can be as low as 1.16%. 4 VTEs result in significantly higher health care costs 5 and compromise the heath of the patient. 6 7

National Institute for Clinical Excellence (NICE) in 2010 recommend all patients undergoing lower limb arthroplasty be assessed for VTE risk. 8 Their guidelines recommend offering mechanical and pharmacological prophylaxis from admission in orthopedic surgical patients, but does not include aspirin. 8 The pharmacological prophylaxis should continue for 28 to 35 days for hip surgeries, and 10 to 14 days for knee surgeries as a minimum. 8 Pharmacological agents recommended does not include aspirin. 8 This contrasts with the American College of Chest Physician (ACCP) guidelines, 2 which recommend aspirin as a potential agent for VTE prophylaxis but state that low molecular weight heparin (LMWH) should be considered first line. They recommend that prophylaxis should be continued for 35 days. 2 The American Academy of Orthopaedic Surgeons (AAOS) guidelines recommend a multimodal approach, but they could not make recommendations on any type of agent and length of treatment due to lack of evidence. 1 They state that these decisions should be made after a discussion between the physician and patient. 1 Therefore, currently all three guidelines do not agree with each other due to different interpretations of the available evidence. This has led to big variations in prescribing of prophylactic agents for VTE, as reported by Cohen et al. 9

## Aspirin as Prophylaxis

Aspirin (acetylsalicylic acid) is an acetylating agent 10 that irreversibly inhibits cyclooxygenase, blocking prostaglandin synthesis and formation of thromboxane A2. 11 Thromboxane A2 aids platelet aggregation. 12

A literature review in 2013 aiming to look at aspirin in DVT prevention in orthopedic surgery, by Drescher et al, 13 looked at eight studies comparing aspirin to anticoagulants. For hip and knee arthroplasty, aspirin performed as well as anticoagulants in the studies they screened, with a reduction in bleeding risk. Another similar review published in 2015 by Sahebally et al 14 found that aspirin appeared as effective as LMWH with a potential for lower bleeding rates, and concluded aspirin could be used as prophylaxis against DVT in those with a high risk of bleeding in orthopedic surgery. Both these reviews looked up to 2013.

The aim of this paper is to review the literature on past 3 years (2014–2017) for the use of aspirin in the VTE prophylaxis in elective lower limb arthroplasty surgery to provide on update.

## Materials and Methods

A MEDLINE and EMBASE search was conducted using key terms including “aspirin” OR “antiplatelet” AND “VTE” OR ”DVT” OR “thromb*” OR “deep vein” AND “orthop*” OR “surgery” OR “operative” OR “prevent*” OR “prophylaxis.” Studies had to be original research, published in peer-reviewed journals; one of the primary outcomes had to be rates of symptomatic DVT; they had to been in English; and published between 2014 and present. Exclusion criteria included if the papers investigated arterial thrombosis, if aspirin was not included, or if the papers did not compare aspirin to another thromboprophylactic. Studies comparing chemical prophylaxis in conjunction with mechanical prophylaxis were included. After the search, the papers were filtered based on their title and abstract, and then the whole paper by D.M. and P.L. The references and related articles of these papers were screened to check for additional papers.

The primary outcome of this review was incidence of DVT in patients prescribed aspirin for VTE prophylaxis hip or knee arthroplasty surgery.

## Results

The literature search produced 212 articles. By screening the titles and abstracts, this number was narrowed down to 52 and 19, respectively. After reading the whole paper and screening their references/related articles, eight pieces of peer-reviewed original research met the inclusion criteria ( Table 1 ).

**Table 1:** Summary of papers found by literature search that meet the inclusion criteria

Study author	Type of study	Total number of patients	Surgery	Follow-up time	Comparison	Aspirin dose	Outcome measure
Raphael et al, 2014 15	Retrospective study	28,923	Total joint arthroplasty	90 d	Aspirin vs. variable dose warfarin	325 mg BD	Symptomatic VTE, complications, mortality.
Jiang et al, 2014 16	RCT	120	Total knee arthroplasty	Unknown	Aspirin vs. LMWH/ rivaroxaban	Unknown	Incidence of VTE
Zou et al, 2014 17	RCT	324	Total knee arthroplasty	Unknown	Aspirin vs. LMWH vs. rivaroxaban	100 mg/day	Incidence of DVT, complications
Na et al, 2015 18	Retrospective study	328	Total knee arthroplasty	3 mo	Aspirin vs. LMWH and aspirin	100 mg/d	Incidence of DVT or PE, complications
Asopa et al, 2015 19	Retrospective study	9,035	Hip and knee arthroplasty	6 wk	n/a	300 mg/d only given on discharge	Incidence of symptomatic VTE
Pow and Vale, 2015 20	Retrospective study	402	Total hip and knee arthroplasty	Unknown	Aspirin vs. anticoagulants in hospital and discharge	Unknown	VTE prophylaxis prescribing, incidence of VTE
Ogonda et al, 2016 21	Retrospective study	11,459	Total hip, total knee, and unicompartmental knee arthroplasty	3 mo	Aspirin vs. National Joint Registry rates	150 mg/d	Incidence of DVT and PE, mortality
Yhim et al, 2017 22	Retrospective study	306,912	Total hip and knee arthroplasty	3 mo	Aspirin vs. anticoagulants and no thromboprophylaxis	Unknown	Incidence of VTE, prescription rates

Abbreviations: BD, twice a day; DVT, deep vein thrombosis; LMWH, low molecular weight heparin; PE, pulmonary embolism; RCT, randomized control trial; VTE, venous thromboembolism.

Raphael et al 15 performed a retrospective data collection study after the change in AAOS guidelines in 2011. 1 They found 28,923 who had undergone total joint arthroplasty, of which 2,800 has received aspirin, and looked at whether they had symptomatic VTE, wound complications, and mortality up to 90 days postsurgery. Patients were on either 325 mg aspirin twice a day or variable dose warfarin with an international normalized ratio (INR) range of 1.5 to 1.8. The incidence of symptomatic pulmonary embolism (PE) and DVT in the aspirin group was lower than in those on warfarin, 0.29% compared with 0.99%, and was statistically significant. Patients given aspirin had a shorter stay in hospital and less wound-related problems. Raphael et al 15 concluded that aspirin is safe, “well-tolerated, inexpensive, and easy to administer” and they wanted future prospective studies to be performed into thromboprophylaxis in arthroplasty patients, to determine safer and effective protocols.

A randomized control trial (RCT) in China 16 compared aspirin with LMWH and rivaroxaban in 120 patients undergoing total knee replacement. Of the 60 patients prescribed aspirin and 60 prescribed anticoagulants, zero were diagnosed with DVTs. Their analysis showed aspirin was as effective as anticoagulants and had a better safety profile—less blood loss and less subcutaneous ecchymosis. There was also a proven cost reduction with using aspirin compared with LMWH and rivaroxaban.

Another RCT by Zou et al 17 looked at 324 patients undergoing knee arthroplasty and administered either aspirin (100 mg/day), rivaroxaban, or LMWH. Treatment lasted 14 days. There were two symptomatic DVTs in the aspirin group, one was treated with LWMH, and none in the rivaroxaban group. The rivaroxaban group had the highest incidence of would complications, bleeding, and subcutaneous ecchymosis. While it was out-performed by rivaroxaban, there were no statistically significant differences between aspirin and LMWH in efficacy and it had the best safety profile of the three. Zou et al 17 argued that aspirin, when used with other methods of thromboprophylaxis (such as mechanical), may be effective in prevention of VTE after knee arthroplasty, and be preferred over anticoagulants.

A Korean study by Na et al 18 looked at the 328 patients. Patients were prescribed thromboprophylaxis based on their risk of VTE and bleeding, which was assessed using recommendations from the AAOS guidelines. All patients were treated for 14 days postoperatively. Patients with a high risk of VTE and standard risk of bleeding were given enoxaparin 40 mg for 7 days, then aspirin 100 mg once daily. Those with elevated risk of bleeding and normal risk of VTE were given antiembolic elastic stockings and those with increased or standard risk of both were given aspirin 100 mg once daily. In total, 282 patients were given aspirin. They found that the risk of VTE in Korean patients is low; and backed the risk stratification guidelines they used. No patients in this study suffered from a symptomatic DVT or PE. Note that 15.5% of patients experienced minor bleeding complications and 10.7% of patients stopped chemical prophylaxis. The lack of any symptomatic DVTs in Na et al's 18 study showed the regimes they used were effective at preventing DVTs. However, due to low rates of VTEs in Korean patients, they could not conclude that chemical thromboprophylaxis was necessary.

A retrospective study by Asopa et al 19 looked at 9,035 patients undergoing hip and knee arthroplasty; investigating the VTE rate of its current protocol. Those deemed high risk were given 20 mg LWMH twice a day until discharge and then aspirin 300 mg once daily. The low-risk patients were given 20 mg LWMH once daily in hospital, and then aspirin 300 mg once daily. All patients were followed up at 6 weeks. Total VTE rate was 2.55% (230 cases). There were reports of complications including wound oozing, wound infection, hematoma, hemarthrosis, and wound dehiscence in 363 individuals. They stated their data showed that staged prophylaxis using LMWH and aspirin was good at preventing VTEs; however, due to complications experienced, a safer protocol could be developed.

Pow and Vale's 20 study in one private hospital in Australia looked at 400 patients undergoing total hip and knee arthroplasty, who received chemical thromboprophylaxis for a minimum of 10 to 14 days. Almost half received LMWH and 125 received aspirin while in hospital. Aspirin was the most commonly prescribed thromboprophylactic agent on discharge, and on average prophylaxis was prescribed for 22 days. Wound oozing was reported as more common in those on anticoagulants and there were two episodes of minor bleeding. The rate of VTE was 4.7% (19), 18 of which were DVTs. It was reported that there were no significant differences between the incidence of VTEs in patients prescribed with anticoagulants, compared with those prescribed antiplatelets. Four patients out of 125 on aspirin had a VTE, 15 out of 275 on an anticoagulant agent—the one patient who developed a PE was on warfarin. Pow and Vale 20 concluded that the rates of VTE in their study were high due to the length of the thromboprophylaxis regime. They stated good risk stratification and implementation of a better, multimodel protocol for thromboprophylaxis is required in their center.

Ogonda et al 21 retrospectively looked at data on almost 12,000 patients undergoing arthroplasties of the hip and knee spanning from 2002 to 2014, specifically looking at the incidence of DVTs/PEs and mortality. Their protocol involved giving aspirin 150 mg once daily to these patients for 6 weeks starting on the first postoperative day. All patients were mobilized on the first day too and patients treated after 2012 were also given mechanical prophylaxis, unless contraindicated. Their results showed an incidence of 0.32% across all procedures over the 13-year period. Aspirin also did not increase 90-day mortality and there was not an increase in VTE incidence in these patients either, when compared with the National Joint Registry for England and Wales. They concluded that aspirin is safe to use in hip and knee arthroplasty as thromboprophylaxis, but a prospective RCT should be performed in the future.

Using a government-affiliated organization's database in Korea, Yhim et al 22 looked through 306,912 patient's data after they had undergone knee or hip arthroplasty between 2009 and 2013. Note that 57.6% of patients were treated with chemical thromboprophylaxis, for 9 days on average. Only 9.18% of patients (28,176) were treated with aspirin. They reported that incidences of VTE were higher in those receiving thromboprophylaxis, compared with those not receiving it. Of those receiving aspirin, 454 patients had VTEs—232 had DVTs alone. The rates of VTE in those taking aspirin were lower than those of LMWH, similar to those of rivaroxaban and only slightly higher than those of fondaparinux. Aspirin did not increase the need for blood transfusions unlike other anticoagulants.

### Result Summary

Seven of the eight studies gave suitable information and can be found in Table 2 , which looks at the rates of symptomatic DVTs in patients prescribed aspirin ( Table 2 ).

**Table 2:** Comparison of patients on aspirin in the arthroplasty studies with the number and rates of VTE

Study author	Dose of aspirin	Length of treatment	Number of patients on aspirin	Number of patients who suffered from symptomatic DVT	Rate of symptomatic DVT
Raphael et al, 2014 15	325 mg BD	Unknown	2,800	8	0.29%
Jiang et al, 2014 16	?	?	60	0	0.00%
Zou et al, 2014 17	100 mg/d	14 d	110	2	1.81%
Na et al, 2015 18	100 mg/d	14 d	282	0	0.00%
Pow and Vale, 2015 20	Unknown	Minimum 10–14 d	125	4	3.20%
Ogonda et al, 2016 21	150 mg OD	6 wk	11,459	37	0.32%
Yhim et al, 2017 22	Unknown	On average for 9 d	28,176	232	0.82%
Total	−	−	43,012	283	0.66%

Abbreviations: BD, twice a day; DVT, deep vein thrombosis; OD, once daily; VTE, venous thromboembolism.

The total rate of symptomatic VTEs from the table above is 0.66% in over 43,000 patients treated with aspirin for thromboprophylaxis.

## Discussion

The ACCP stated that symptomatic VTE rates can be as high as 4.3% 2 and it has been reported that the rate of symptomatic DVT can be 1.16%, despite prophylaxis. 4 Of the eight studies looked at in this report, a DVT rate of 0.66% was found with five of them backing aspirin as prophylaxis for VTE after orthopedic surgery 15 16 17 18 21 and the other three showing promising results. 19 20 22 It is surprising therefore that NICE still do not recommend aspirin as VTE prophylaxis after major lower limb orthopedic surgery. 8

Ogonda et al 21 and Yhim et al 22 looked at the most patients on aspirin; both had the lowest rates of DVT. These studies justify future prospective studies to be performed into thromboprophylaxis in arthroplasty patients, to determine safer and effective protocols.

Asopa et al 19 stated that their LMWH dose was subtherapeutic due to bleeding concerns, when the protocol was made. This highlights one of the key issues with LMWH—bleeding complications. Problems with this study are that they only followed up patients for 6 weeks as opposed to the majority of other studies, which followed up patients for 3 months. Another limitation is that the authors did not perform the risk stratification themselves (so had to rely on another clinician's data); this is a problem with retrospective studies. Its use of a mixed regime meant it was unsuitable for comparing the rates of DVTs seen in Table 2 .

Pow and Vale 20 could not support aspirin outright as a thromboprophylactic agent due to the suboptimal prophylactic regime investigated in their study (a quarter of patients were not prescribed thromboprophylaxis after discharge). This is despite there being no significant difference in the incidence of VTEs in those receiving aspirin and those receiving anticoagulants. The regime was unfortunately something they could not control, an issue with a retrospective study.

A review study in 2014 13 comparing aspirin with other anticoagulants found no difference between aspirin and other anticoagulants in arthroplasty surgery—a conclusion supported by many papers in this study. Sahebally et al 14 suggested that aspirin is an effective thromboprophylactic and calls for more studies comparing aspirin and anticoagulants. The conclusions from recent systemic reviews, Drescher et al 13 and Sahebally et al, 14 and this study suggested that aspirin is not inferior to anticoagulants.

A huge benefit of aspirin is the safety profile, which was confirmed yet again by six of the studies. Aspirin is cost-effective and does not require monitoring, so is well tolerated. 23 This may be one contributing factor as to why aspirin prescribing for VTE prophylaxis after arthroplasty has increased. 24 They also stated that the change to the AAOS and ACCP guidelines helped increase this too. 24

Common themes running throughout the studies were the importance of early mobilization, with some studies saying patients should be mobilized on the first postoperative day. Some, including Ogonda et al, 21 combined aspirin with mechanical prophylaxis; and others even employed a risk stratification system before selecting the type of thromboprophylaxis. These points are recommended for all patients undergoing major lower limb orthopedic surgery by the AAOS 1 and ACCP 2 guidelines.

Regarding the Korean papers, it is mentioned that in the Asian population, there are lower rates of DVT, 18 25 26 which explains why VTE prophylaxis is not routinely prescribed in Korea. 27 This may be why Yhim et al 22 found no statistically significant difference between the rates of DVTs in those prescribed aspirin and those prescribed no thromboprophylaxis. Yhim et al 22 did use a large national database; the data may not be accurate and the quality of data are often poor due to the issue of data capture. It may be difficult to compare the results from Korean populations with Caucasian populations.

Bleeding is a complication of thromboprophylaxis; this effect is much more profound in nonaspirin chemical anticoagulants. Two studies investigating the safety profile of warfarin and LMWH found that major bleeding rates with warfarin were 4.7 and 7.9%; and with LMWH it was 3.9%; 28 29 and one person died from massive gastrointestinal (GI) bleed while on warfarin. 28 Other oral anticoagulants, like rivaroxaban, are also susceptible to these complications as shown by Jiang et al 16 and Zou et al. 17

Limitations of this study include that the evidence reviewed only focused on aspirin; a comparison between aspirin and a particular anticoagulant could have been performed. As this report looked at 2014 to 2017, it could be argued that the scope of evidence was limited; however, Drescher et al 13 and Sahebally et al 14 summarized previous evidence effectively. Unfortunately, not all papers mentioned the dose, timing, and length of prophylaxis, and one study did not give suitable information for about the rates of DVTs in patients prescribed aspirin.

## Conclusions

This study has suggested that there is evidence to support aspirin as thromboprophylaxis for VTE following major elective lower limb orthopedic surgery, which is backed up by its inclusion in the ACCP and AAOS guidelines. NICE should revise their dated guidelines, taking into account the evidence presented in this review. The comparison of studies in this review found a rate of 0.66% of symptomatic DVTs in patients who were prescribed aspirin after arthroplasty surgery. Aspirin is not only effective but has a good safety and side effect profile, especially when compared with other oral anticoagulants.
